# Gestational weight gain and fetal growth in underweight women

**DOI:** 10.1186/s13052-016-0284-1

**Published:** 2016-08-05

**Authors:** Vincenzo Zanardo, Alessandro Mazza, Matteo Parotto, Giovanni Scambia, Gianluca Straface

**Affiliations:** 1 Division of Perinatal Medicine, Policlinico Abano Terme, Piazza Colombo 1, 35031 Abano Terme, Italy; 2 Department of Anesthesia, University of Toronto, Toronto, ON Canada; 3 Department of Obstetrics and Gynecology, Catholic University of sacred Heart, Rome, Italy

**Keywords:** Underweight women, Pre-pregnancy body mass index, BMI, Gestational weight gain, GWG, Fetal growth

## Abstract

**Background:**

Despite the current obesity epidemic, maternal underweight remains a common occurrence with potential adverse perinatal outcomes.

**Methods:**

We aimed to investigate the relationship between weight gain during pregnancy, and fetal growth in underweight women with low and late fertility. Women body mass index (BMI), defined according to the World Health Organization’s definition, gestational weight gain (GWG), defined by the Institute of Medicine and National Research Council and neonatal birth weight were prospectively collected at maternity ward of Policlinico Abano Terme (Italy) in 793 consecutive at term, uncomplicated deliveries.

**Results:**

Among those, 96 (12.1 %) were categorized as underweight (BMI < 18.5 kg/m^2^), 551 (69.5 %) as normal weight, 107 (13.4 %) as overweight, and 39 (4.9 %) as obese, respectively. In all mother groups, GWG was within the range recommended by IOM 2009 guidelines. However, underweight women gained more weight in pregnancy (12.8 ± 3.9 kg) in comparison to normal weight (12.3 ± 6.7 kg) and overweight (11.0 ± 4.7 kg) women and their GWG was significantly higher (*p* < 0.001) with respect to obese women 5.8 ± 6.1 kg). In addition, offspring of underweight women were comparable in size at birth to offspring of normal weight women, whereas they were significantly lighter to offspring of both overweight and obese women.

**Conclusions:**

Pre-pregnancy underweight does not impact birth weight of healthy, term neonates in presence of normal GWG. Presumably, medical or personal efforts to reach ‘optimal’ GWG could be a leading choice for many women living in industrialized and in low-income countries.

## Background

It has been explicitly recognized that both pre-pregnancy body mass index (BMI) and gestational weight gain (GWG) may influence pregnancy outcome and offspring birth weight [1, 2]. It has been nearly two decades since guidelines for how much weight a woman should gain during pregnancy were issued by the Institute of Medicine (IOM) [3]. In that time, more research has been conducted on the effects of weight gain in pregnancy on the health of both mother and baby. There have also been dramatic changes in the population of women having babies. This is relevant, considering that despite the current obesity world-wide epidemic, at the other end of the spectrum, maternal underweight, defined according to the World Health Organization’s (WHO) definition of body mass index (BMI) <18.5 kg/m^2^ is also common [4]. For instance, at the first antenatal visit 4.3 % of pregnant women in the UK [5] and 9.0 % of women in China [6] are underweight according to the WHO definition. Moreover, while the vast majority of women in Pakistan embark on pregnancy undernourished [7], a population-based Swedish study observed that 9.6 % of women had a BMI in the range of 15–19.9 kg/m^2^ [8].

When BMI-specific ranges for “optimal” weight gain were introduced by IOM [9], the nature of the combined effects of pre-pregnancy BMI and GWG on birth weight has been considered in more detail. Adequate pre-pregnancy weight and weight gain during pregnancy were associated with better neonatal and maternal outcomes [3]. In particular, several studies have ascertained the association between low pre-pregnancy BMI and inadequate GWG and low birth weight [2, 6]. Conversely, it has been suggested that obese women may benefit from low GWG [4]. However, understanding these associations is also complex, because BMI is closely linked to lifestyle factors, diseases, and genetic traits that are also correlated with maternal, physiological and social characteristics [8]. In addition, GWG may be also variable and modifiable during gestation or associated with a poor nutritional status and its timing change has an impact on birth weight [2, 10, 11]. In addition, limited literature is available on the pattern and perinatal consequences of pre-pregnancy BMI and GWG of women from industrialized countries, based on BMI-specific ranges for “optimal” weight gain introduced by IOM in 2009 [12–14]. Pre-pregnancy BMI and GWG are key parameters to distinguish a low pre-gestational BMI determined from an aesthetic choice and the research of an ideal pre- and in-pregnancy idealized body shape from a pour nutritional status or from a global in utero impaired growth.

Hence, there is a need to see the contextual relevance of these determinants to our own population, representative of developed, industrialized countries supporting advanced educational levels, good socio-economic status, and low and late fertility. We therefore undertook an analytical study with a reference group of pre-gestational low BMI women to determine the direction and magnitude of the effect of maternal underweight and GWG on fetal growth in singleton, at term, uncomplicated pregnancies.

## Methods

This prospective study was performed at the maternity wards of the Division of Perinatal Medicine of Policlinico Abano Terme, Abano Terme, Italy. The hospital where the study took place is located in an industrialized area supporting advanced educational levels, good socio-economic status, occupation, and low and late fertility.

The study group consisted of 793 consecutive mothers of singleton, healthy neonates delivered at term. Women that didn’t speak Italian, underwent general anaesthesia, with psychological treatment and with infants hospitalized in NICU, were excluded from the study.

After informed consent was obtained, they answered sociodemographic questions before and after pregnancy, obstetric history, smoking, psychological care, plan to lactate an infant without medical or obstetrical complications precluding routine discharge.

It was an analytical study with the use of secondary data [15]. The study variables were taken from the database of an ongoing prospective large cohort study conducted on symptoms of eating disorders in pregnancy [15]. Institutional Review Board (Policlinico Abano Terme) approval was obtained before the study began.

Every singleton at term (>37 < 42 weeks) pregnancy with known GWG indicators and maternal and neonatal outcomes were eligible. After informed consent was obtained, they answered sociodemographic questions before and after pregnancy, obstetric history, smoking, psychological care, plan to lactate an infant without medical or obstetrical complications precluding routine discharge. The following data were also collected for the babies: gestational age, birth weight, length, head circumference, and mode of delivery.

Pre-pregnancy weight was used as recommended by other studies on gestational weight gain [13–15]. BMI was calculated by using the standard formula of person’s weight in kilograms divided by the square of his height in meters (kg/m^2^). The women were categorized into four categories with respect to their BMI as per the standard of institute of Medicine [9] as underweight (BMI < 18.5 kg/m^2^), normal (BMI ≥18.5 and < 25 kg/m^2^), overweight (BMI ≥25 and <30 kg/m^2^), and obese (BMI ≥30 kg/m^2^). Gestational age was recorded by last menstrual period and verified by ultrasound as a routine practice within the institution. Total weight gain was calculated by subtracting the pre-pregnancy weight from the last measured weight before delivery and ‘optimal’ ranges were categorized according to 2009 IOM guidelines: underweight 12.5–18 kg, normal weight 11.5–16 kg, overweight 7–11.5 kg, and obese 5–9 kg, respectively [9]. Fetal growth was estimated at birth, noting birth weight, length, and head circumference. The effect of gestational weight gain on adverse fetal growth were compared as per guidelines from the institute of Medicine [9], while controlling for BMI, parity, delivery mode, maternal age, educational level, and working status. To determine the association of adverse pregnancy outcome with pre-pregnancy BMI and gestational weight gain according to the IOM recommendations chi-square test and student *t* test were used at 0.05 level of significance. Statistical analyses were carried out using Statistical Package for Social Sciences (SPSS) version 19 (IBM, Armonk, NY).

## Results

Anthropometrical and clinical features of the study population are shown in Table 1.

**Table 1:** Anthropometrical and clinical characteristics of the study population

Pre-gestation women, n.	793
Age, years	33.0 ± 4.7
Height, cm	165.1 ± 5.9
BMI, kg/m^2^	22.4 ± 4.1
Smoking	29 (3.6)
Educational level:	
Junior high school	98 (12.3)
High school	413 (52.0)
University degree	276 (34.8)
Working status:	
Working	607 (76.5)
Housewife	101 (14.0)
Student	14 (1.7)
Unemployed	49 (6.1)
Parity:	
Nulliparous	446 (56.2)
2	255 (32.1)
> 2	75 (9.4)
Cesarean section:	214 (26.9)
Elective	111 (13.9)
Vacuum extractor	25 (3.1)
Fetal Growth:	
Weight, g	3402.5 ± 411.5
Length, cm	50.1 ± 1.7
Head Circumference, cm	34.3 ± 3.2
LGA (>4000 g)	51 (6.4)
NICU admission	3 (0.3)

Data expressed as Mean ± SD or percent (%)

This cohort study is characterized by advanced educational levels, good socio-economic status, occupation, and low and late fertility. Women were 33.0 ± 4.7 years old, 56.2 % were nulliparous, most completed high school (52.0 %) or graduated from university (33.5 %), and 82.2 % were employed, and 9 (7.8) 49 (6.1) were unemployed. Smoking behaviour was present in 3.6 %. Only 3 neonates, 1 offspring of a normal weight woman and 2 of overweight women, respectively, were NICU admitted (1 GBS sepsis, 1 labiopalatoschisis, and 1 intestinal malrotation).

Anthropometrical and clinical characteristics of the mother-infant dyads categorized according to the Institute of Medicine [9] BMI and GWG guidelines is reported in Table 2.

**Table 2:** Anthropometrical and clinical characteristics of the mother-infant dyads categorized according to the Institute of Medicine [9] guidelines

Pre-pregnancy BMI (kg/m^2^):	<18.5	≥18.5 < 25	≥25 < 29	≥30
	Underweight	Normal	Overweigh	Obese
	96 (12.1)	551 (69.4)	107 (13.4)	39 (4.9)
n. 793	96 (12.1)	551 (69.5)	107 (13.5)	39 (4.9)
Age, years	31.9 ± 5.1	33.4 ± 4.7	32.8 ± 4.7	32.0 ± 5.2
Height, cm	166.7 ± 6.0	165.2 ± 5.8	164.8 ± 6.1	163.7 ± 6.0
BMI, kg/m^2^	17.6 ± 0.7	21.4 ± 1.6	26.7 ± 1.3	33.3 ± 3.9
GWG, kg	12.8 ± 3.9	12.3 ± 6.7	11.0 ± 4.7	5.8 ± 6.1^#^
Parity:				
Nulliparous	51 (53.1)	321 (58.2)	54 (50.4)	20 (51.2)
2	31 (32.2)	165 (29.9)	45 (42.0)	14 (35.9)
> 2	9 (9.3)	55 (9.9)	7 (6.5)	4 (10.2)
Smoking	6 (6.2)	16 (2.9)	5 (4.6)	2 (5.1)
Educational level:				
Junior high school	11 (11.4)	66 (11.9)	15 (14.0)	6 (15.3)
High school	51 (53.1)	285 (51.7)	54 (504)	23 (58.9)
University degree	31 (32.2)	192 (34.8)	35 (32.7)	8 (20.5)
Working status:				
Student	1 (1.0)	8 (1.4)	4 (3.7)	1 (2.5)
Working	82 (85.4)	428 (77.6)	80 (74.7)	17 (43.5)
Housewife	4 (4.1)	79 (14.3)	17 (15.8)	11 (28.2)
Unemployed	9 (9.3)	36 (6.5)	4 (3.7)	0
Cesarean section:	16 (16.6)	153 (27.7)	31 (28.9)	14 (35.8)
Elective	7 (7.2)	79 (14.3)	20 (18.6)	5 (12.8)
Vacuum extractor	2 (2.5)	19 (4.7)	3 (3.9)	1 (4.0)
Fetal Growth:				
Weight, kg	3239.2 ± 423.5	3264.0 ± 421.9	3422.7 + 471.2*	3458.6 ± 414.1**
Length, cm	49.5 ± 1.9	49.9 ± 1.9	50.0 ± 2.9°	50.8 ± 1.7°°
Head Circumference, cm	33.8 ± 1.3	34.1 ± 1.7	34.5 ± 2.5^	34.5 ± 1.8^^
LGA	2 (2.0)	33 (5.9)	12 (11.2)	4 (10.2)

Data expresses as Mean ± SD or percent (%)

*BMI* Body Mass Index, *GWG* Gestational Weight Gain

^#^ *p* < 0.001 vs. underweight

*p < 0.001 vs. underweight; **p < 0.01 vs. underweight

°*p* < 0.001 vs. underweight; °°*p* < 0.003 vs. underweight

^p < 0.02 vs. underweight; ^^p < 0.02 vs. underweight

Among 793 consecutive women included in this analysis, 96 (12.1 %) were categorized as underweight (Mean ± SD, BMI, 17.6 ± 0.7), 551 (69.5 %) as normal weight, 107 (13.4 %) overweight, and 39 (4.9 %) obese, respectively.

Underweight women were 31.9 ± 5.1 years old, 53.1 % were nulliparous, most completed high school (53.1 %) or graduated from university (32.2 %), and 85.4 % were employed, and 9.3 % were unemployed. Smoking behaviour was 6.2 %.

In all mother groups, GWG was within the range recommended by IOM 2009 guidelines. However, underweight women gained more weight in pregnancy (12.8 ± 3.9 kg) in comparison to normal weight (12.3 ± 6.7 kg) and overweight (11.0 ± 4.7 kg) women and their GWG was significantly higher (*p* < 0.001) with respect to obese women 5.8 ± 6.1 kg).

Our results have also shown that offspring of underweight women were comparable in size at birth to offspring of normal weight, but they were significantly lighter to offspring of both overweight and obese women.

Finally, underweight women have shown almost halved rates of large for gestational age neonates (LGA) (2.0 vs. 5.9 %) and of operative delivery, caesarean section (16.6 vs 27.7 %), and vacuum extractor (2.5 vs. 4.7 %), respectively, in comparison to normal BMI women. The above-described reduced rates appeared also more pronounced with respect to overweight and obese women.

## Discussion

Following IOM BMI-specific ranges for “optimal” weight gain in women categorized by weight group, we explored the impact of the combined effects of pre-pregnancy low BMI and GWG on fetal growth, in an urban setting of women living in an industrialized area of North-Eastern Italy. In this cohort study, inclusive of women with advanced educational levels, good socio-economic status, and low and late fertility, pre-pregnancy low BMI was common and involved more than 1:10 women, whereas mean GWG was within the range recommended by IOM 2009 guidelines in this and in the other weight groups.

Our results have also shown that offspring of underweight were comparable in size at birth to offspring of normal weight women, and significantly lighter than offspring of both overweight and obese women. However, occurrence of LGA neonates and operative delivery rate, caesarean section and vacuum extractor, were almost halved in underweight women in comparison to normal weight women and even three-four times lower in comparison to underweight or obese women.

One of the most important modifiers of pregnancy weight gain and its impact on a mother’s and her baby’s health is a woman’s weight at the start of pregnancy [16]. However, few studies have considered the impact of taking into account both pre-pregnancy low BMI and GWG, on the quantification of the association on fetal growth in developed countries after the introduction of the 2009 IOM guidelines that defined ‘optimal’ GWG [4]. This is relevant, considering that women in the Western world are heavier than ever, and being pregnant contributes further to obesity [17, 18]. Moreover, despite the current obesity world-wide epidemic, at the other end of the spectrum, maternal underweight, is also common for opposite reasons in low income as well as in industrialized countries [10].

The underweight prevalence in this study is similar to that reported in a Chilean women population [19] and consistent with other investigations in China (9.0 %) [6] and in Sweden (9.6 %) [4], but is much higher than that reported in the UK population (4.3 %) [5]. Not surprisingly, BMI <18.5 is about 70 % in India, Bangladesh and Pakistan [20]. For Africa, this proportion is 20–40 % [21]. What is novel in this Italian cohort study, is that underweight women had a mean weight gain within the IOM guidelines range, and that this was without deleterious consequences for themselves or their infants, differently from what reported in underweight, disadvantaged women living in low income countries. Ethnicity and socio-economic status in itself may be responsible for these differences. Among the factors contributing to poor outcomes in mothers and their infants from disadvantaged backgrounds are inadequate dietary and nutritious intakes due to poor access to health care, limited or no knowledge about nutritious diet in pregnancy, and use of prenatal vitamins [22, 23]. Along with poverty, strong cultural norms play vital role in determining women’s health pre- and during pregnancy [24]. This accentuates an immense need of antenatal education for pregnant women of all socio-economic and demographic class, so that women with different pre-pregnancy BMIs can have a healthy pregnancy if ‘optimal’ GWG is guaranteed. We could speculate that medical management and/or personal efforts for not gaining too much weight could be responsible for a part of this association. Possibly, during pregnancy, fat is stored to secure energy supply during fetal growth and lactation. In obese women, no additional storage is necessary, which suggests that pregnancy weight gain could be restricted because of physiological mechanisms. Total weight gain during pregnancy remains variable and is influenced by a number of factors. It is relevant that weight gain in pregnancy is lower when pre-pregnancy BMI is higher, and in this cohort study the mean weight gain during low BMI pregnancy was 12.8 + 3.9 kg, which is higher (5.8 ± 6.1 kg) to that registered in obese women, but within the range recommended by IOM guidelines.

Understanding these associations is also complex, because pre-gestational BMI and GWG, considered predictors of the maternal nutritional status, are closely linked to lifestyle, prevalent body image models, and socio-cultural factors in industrialized countries and, in contrast, to endemic malnutrition, diseases, and genetic traits in disadvantaged, low income countries [25]. In addition, when the Institute of Medicine (IOM) last revised the recommendations for weight gain during pregnancy [3] it emerged that a woman’s nutritional status at conception may modify not only the course of pregnancy and its outcome but also the way her nutritional status changes during pregnancy [26, 27]. Noteworthy, GWG timing change has an impact on pregnancy outcomes, (e.g., preterm birth, IUGR, or LGA neonates), which also complicates the interpretation of these relations (e.g., operative delivery, caesarean section). Thus, whether maternal underweight and/or low GWG is associated with increased risk, decreased or neutral risks of preterm birth is still debated in the literature [28, 29]. Preterm birth persists as the leading cause of neonatal morbidity and mortality [30–32].

After excluding cases of abnormal GWG, a stronger similitude between fetal growth of offspring of underweight and normal weight women appeared. This is the first time this result has been reported, and it suggests that the known relationship between pre-gestational BMI and offspring birth weight obscures, in part, the association between pre-pregnancy underweight or low GWG and birth weight previously reported in low income women, and this must be taken into account. It is possible that the ‘optimal’ weight gain in women with low BMI may have received less attention from clinicians but may indeed play a strong role in the risk of LBW and adverse pregnancy [33].

Although we adopted a reproducible method to classify maternal BMI, GWG and fetal growth, we were not able to exclude some other limitations of this study, which therefore need to be addressed. Firstly, the relevant correlation between birth weight and gestational age of the newborns is lacking. This information could have some impact on postnatal adverse health outcomes (i.e., low Apgar score and/or NICU admission) [6, 34]. Another limitation of this study is that it was conducted in a single center of North-Eastern Italy, nor takes into account regional specificities of way of life and obstetrical practices. We acknowledge that excluding women unable to speak the national language also limits the possibility to generalize our results to the whole high income pregnant women population.

## Conclusion

Our analysis using net maternal weight gain in underweight women living in an industrialized, high income country does not reinforce the association between low BMI and deleterious consequences for themselves or their infants, differently from that reported in underweight, disadvantaged women living in low income countries. Offspring of underweight and normal weight women were comparable in size at birth to offspring of normal weight women and operative deliveries rates, caesarean section and vacuum extractor, were reduced in comparison to normal weight women and even more so in comparison to overweight or obese women. The findings of this study highlight the need for nutritional education and life style adjustments to improve pregnancy outcomes.

## Abbreviations

BMI, body mass index; GWG, gestational weight gain; IOM, the Institute of Medicine; IUGR, intra uterine growth retardation; LGA, large for gestational age; WHO, World Health Organization
